# Morphological and Phytochemical Characterization of Old Ligurian Basil Accessions: Recovery of Old Biodiversity for Future Exploitation

**DOI:** 10.3390/plants14040553

**Published:** 2025-02-11

**Authors:** Federica Betuzzi, Denise Campioli, Paola Malaspina, Fabio Rapallo, Giovanni Bottino, Gloria Scrigna, Giovanni Minuto, Laura Cornara

**Affiliations:** 1 Department of Earth, Environment and Life Sciences (DISTAV), University of Genova, Corso Europa 26, 16132 Genova, Italy; federica.betuzzi@edu.unige.it (F.B.); denise.campioli@rivlig.camcom.it (D.C.); 2 Center for Agricultural Experimentation and Assistance (CeRSAA), Regione Rollo 98, 17031 Albenga, Italy; gloria.scrigna@rivlig.camcom.it (G.S.); giovanni.minuto@rivlig.camcom.it (G.M.); 3 Department of Economics (DIEC), University of Genova, Via F. Vivaldi 5, 16126 Genova, Italy; fabio.rapallo@unige.it; 4 Consorzio di Tutela del Basilico Genovese DOP, Salita Santa Caterina 50-52R, 16123 Genova, Italy; gianni.bottino@basilicogenovese.it

**Keywords:** *Ocimum basilicum*, Liguria, agrobiodiversity, germplasm conservation, glandular trichomes, VOCs

## Abstract

Since the 19th century, the cultivation of *Ocimum basilicum* L. has increasingly been established in Liguria, with the in situ reproduction of seeds. Over the years, Ligurian basil accessions were crossed with allochthonous genomes to obtain disease-resistant plants. To preserve the original genetic resource, nine old Ligurian accessions (CV1–9) were recovered. As part of the PSR 2014–2022 Mis. 10.2 of Liguria Region, this work aimed to characterize these CVs by morphological and phytochemical analyses to safeguard their biodiversity. Commercial *O. basilicum* Genovese Superbo grown in Liguria (SL) was added for comparison. The micro-morphological investigation showed significantly different trichome densities among the samples. CV4 showed the highest densities of both peltate and capitate trichomes, while CV9 and CV1 had the lowest peltate and capitate densities, respectively. In addition, to perform the germplasm characterization, seed morphometric data and germinability were evaluated. Volatile Organic Compounds (VOCs) analysis was carried out on CV1–9, SL, and Superbo plants grown in Piedmont (SP), to test the influence of territory on basil aromatic profiles. The results showed that the old accessions and SL were rich in linalool, eugenol, and bergamotene. Only CV1 slightly differed, with higher levels of methyl eugenol, eucalyptol, and camphor. On the contrary, SP had very high levels of methyl eugenol and camphor. These data represent valuable insights for preserving Ligurian old basil accessions and maintaining the production of Genovese Basil PDO (Protected Designation of Origin) in the future.

## 1. Introduction

*Ocimum basilicum* L. is an herbaceous, annual plant native to the African continent and the Indian subcontinent, belonging to the Lamiaceae family. It was introduced to the Mediterranean basin, and particularly to Italy, between the 17th and 18th centuries. Starting from the 19th century, basil cultivation has increasingly been established in the Liguria Region (Italy), especially to the west of Genoa [1]. In the 1970s, basil production gradually expanded to other Ligurian areas, thanks to the climate, the skills of growers, and the introduction of greenhouse cultivation. This allowed the production of basil both for fresh consumption and for seed propagation.

The basil production cycle was closed, as it involved in situ reproduction by seeds selected over time to preserve the best aromatic characteristics. However, at the end of the 1980s, phytosanitary problems (e.g., the spread of *Fusarium oxysporum* f. sp. *basilici* in the growing areas) and related economic needs led to the opening of this closed system and the search for resistant, or at least disease-tolerant, plants from allochthonous genetic lines. Thus, specialized seed companies improved native seeds, making them more productive and less susceptible to damage from disease attacks [2].

In the following years (1991–1993), the Centre for Agricultural Experimentation and Assistance (CeRSAA) of Albenga (SV, Italy) carried out a census of the areas cultivated with basil and a simultaneous collection of the seeds from some old basil accessions (or cultivars *sensu lato*, CVs) self-propagated by the local farmers. This operation made it possible to recover the following nine local accessions from different areas: 2 in Genoa (GE), 1 in Albenga (SV), 1 in Andora (SV), and 5 in the Dianese zone (IM). Since then, CeRSAA has preserved these seeds, periodically reproducing them using traditional methods, i.e., in a protected environment on soil taken from the area of Albenga and not subjected to disinfection with methyl bromide [3,4].

Over the years, the entire Ligurian territory has demonstrated a high vocation for basil cultivation, obtaining a unique product characterized by an intense and sweet aroma devoid of a minty scent. Therefore, in 2005, basil grown in Liguria has been labelled with the Protected Designation of Origin (PDO) [3], taking the name of “Genovese Basil PDO”, that is renowned worldwide as the key ingredient in pesto sauce. Its characteristics derive from the combined action of the following three factors: the botanical traits of the plant; the environment; and the “human factor”, i.e., the traditional cultivation methods refined over time by Ligurian producers.

According to PDO product specifications [4], the seeds used to produce Genovese Basil PDO must belong to *O. basilicum* derived from native accessions. The plants of Genovese Basil PDO currently on the market adequately express their typical characteristics under the pedo-climatic conditions and traditional growing techniques of Liguria.

Therefore, our work is part of a project financially supported by the Liguria Region (PSR 2014–2022 Mis. 10.2) to study the nine accessions of old basil (called CV1–9) and to ensure germplasm protection and conservation both ex situ (in a repository) and in situ (in growers’ fields), creating a network of “custodians of biodiversity”. In particular, we carried out a multidisciplinary study of these accessions using, as follows: UPOV descriptors (International Union for the Protection of New Varieties of Plants) to define their phenotypical traits; Light and Scanning Electron Microscopy for their micro-morphological characterization; and SPME/GC-MS (Solid Phase Microextraction coupled with Gas Chromatography–Mass Spectrometry) to identify their Volatile Organic Compounds (VOCs) profiles.

The cultivars currently used for producing Genovese Basil PDO are derived from these old accessions (CV1–9). Among the actual commercial cultivars allowed by the PDO specifications, we selected and analyzed Superbo grown in Liguria (SL) for comparison.

For the micro-morphological characterization of all samples, the types and densities of the glandular trichomes scattered on the leaf surfaces were assessed. Moreover, to characterize the germplasm, the germination capacity of seeds and their morphometric parameters were estimated. VOC analysis of all the examined plants was also performed to highlight the more representative compounds emitted by the essential oil (EO), including terpenoids, alcohols, aldehydes, ketones, and esters [5,6]. In addition, VOCs from Superbo grown in Piedmont (SP) were also examined to highlight differences in the aromatic profiles directly linked to the territory of cultivation [7].

This study highlighted the utility of a multidisciplinary approach to characterize old and native crop accessions, thus preserving the agrobiodiversity of typical Italian products. In particular, the aim of our work is to safeguard basil reproduced and cultivated in Liguria for centuries, making it possible to avert genetic erosion and a potential loss of cultural and historical heritage.

## 2. Results

### 2.1. Soil Features

Table 1 describes the composition of the soil, originating from the area of Albenga, used to grow the CV1–9 and SL plants.

### 2.2. Phenotypical Analysis

According to the criteria of the UPOV sheet, the nine old accessions and SL were similar in terms of their phenotypical characteristics. The most significant differences concerned leaf blade color, shape, profile in cross-section, and blistering.

CV1 differed due to a less intense green leaf color, and, together with CV3, due to a concave instead of a convex leaf blade, and an upright blade orientation. CV5, CV6 and CV7 showed a slight leaf blistering, while this trait was absent in the other cultivars. SL was characterized by a darker green leaf color and by a rounded leaf shape instead of lanceolate or ovate leaf shapes.

### 2.3. Micro-Morphological Analysis

#### 2.3.1. Leaves

**Figure 1:**
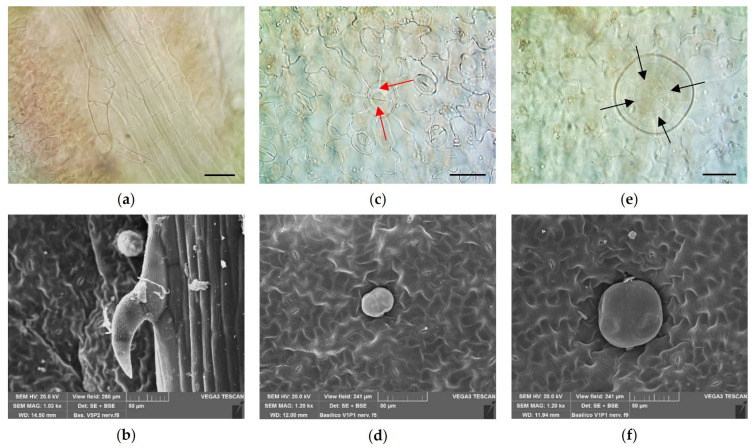
LM (**a**,**c**,**e**) and SEM (**b**,**d**,**f**) micrographs of old basil leaves: (**a**,**b**) an NGT, with a warty cuticle, located on a vein; (**c**,**d**) a CGT with a bicellular head (red arrows); and (**e**,**f**) a PGT with four secretory cells (black arrows), sunken in the epidermis. Bar = 50 micron (**a**), Bar = 30 micron (**c**,**e**).

The micro-morphological investigation showed the distribution of both non-glandular (NGTs) and glandular trichomes (GTs). NGTs were uniseriate, uni- or multicellular, generally bent towards the epidermis, with a tapering apex and a warty surface (Figure 1a,b). They were located only on the leaf veins and along the leaf margin.

Two morphologically distinct types of glandular trichomes were spread on the leaf surfaces: (i) capitate glandular trichomes (CGTs), with a unicellular short stalk and a bicellular head (Figure 1c,d); and (ii) peltate glandular trichomes (PGTs), characterized by a short stalk and a large spherical head with four secretory cells (Figure 1e,f).

**Figure 2:**
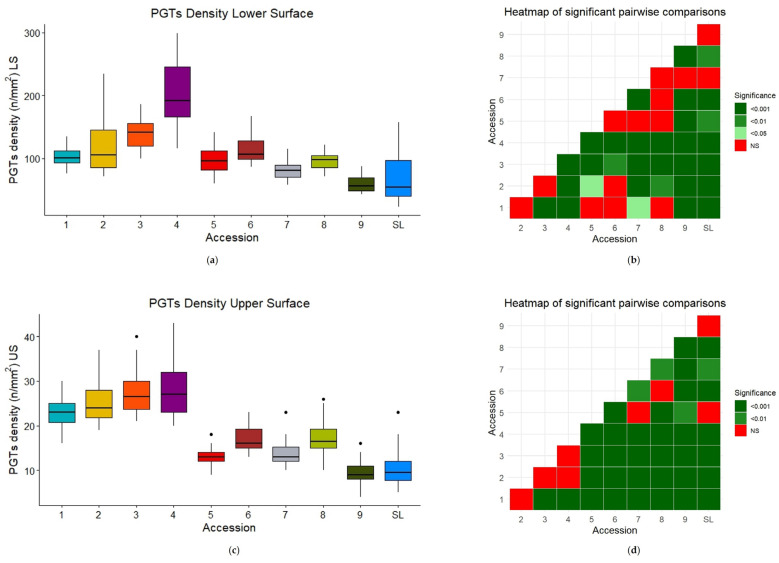
Boxplots of PGTs density on the lower (**a**); and upper (**c**) leaf surface for the nine old accessions (CVs) and SL. Heatmaps [(**b**) and (**d**), respectively] of the pairwise comparisons. Outliers are displayed as individual dots.

**Figure 3:**
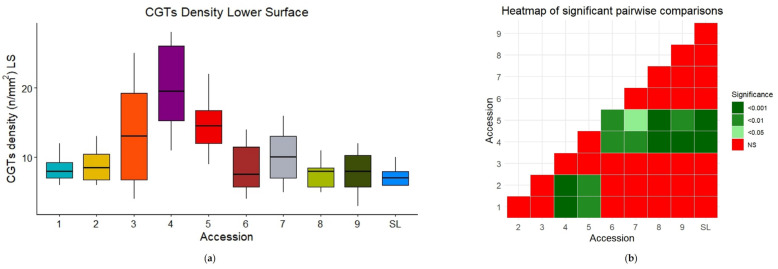

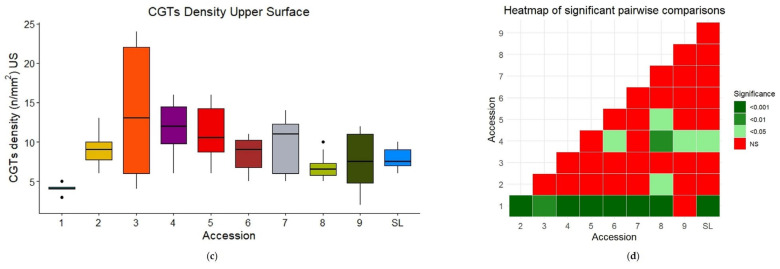
Boxplots of CGTs density on the lower (**a**); and upper (**c**) leaf surface for the nine old accessions (CVs) and SL. Heatmaps [(**b**) and (**d**), respectively] of the pairwise comparisons. Outliers are displayed as individual dots.

Mean values of the GTs count are reported in Tables S1 and S2 in the Supplementary Materials, together with the ANOVA and Kruskal–Wallis results and the post hoc test *p*-values. A graphical representation of the distributions and the heatmap of significant pairwise comparisons are displayed in Figure 2 and Figure 3.

The statistical analysis highlighted that PGT densities were significantly different (*p* < 2 × 10^−16^) between the accessions on both leaf surfaces (lower and upper) (Table S1). In particular, CV4 showed the highest mean PGT density on the lower surface (205.0 ± 52.3), which was significantly different than all other accessions (Table S1, Figure 2a,b). The accessions with the most PGTs on the upper surface were CV2, CV3 and CV4 (Table S1, Figure 2c,d). On the other hand, CV9 had the lowest PGT density on both leaf surfaces (59.6 ± 12.8 and 9.5 ± 2.36 for the lower and upper surfaces, respectively) (Table S1, Figure 2). SL was not significantly different from CV7 and CV9 with respect to PGT density on the lower surface, and from CV5 and CV9 with respect to PGT density on the upper surface (Table S1, Figure 2).

The boxplot of PGT densities on the upper surface (Figure 2c) also revealed that accessions could be divided into two different clusters: a cluster with a mean PGT density < 20, including CV1–4; and a cluster with a mean PGT density > 20, including CV5–9 and SL.

CGT densities were also significantly different (*p* = 4.432 × 10^−7^ for the lower surface, *p* = 8.947 × 10^−7^ for the upper surface) among the accessions (Table S2). CV4 and CV5 showed the highest densities on the lower surface (20 ± 5.92, 15 ± 4.26, respectively) (Table S2, Figure 3a,b), while CV1 had the lowest density on the upper surface (4.08 ± 0.669) (Table S2), also showing a low level of variability (Figure 3c,d). SL differed from CV4 with respect to both surfaces, and only differed from CV5 with respect to the lower surface and from CV1 for the upper one (Figure 3).

In addition, CGT densities were not significantly different between the accessions with respect to different leaf zones that were near the midvein or near the edge (nested two-way nonparametric ANOVA: *p* = 0.363 for the lower surface, *p* = 0.581 for the upper one).

Overall, we observed that PGT densities were higher on the lower surface than on the upper surface. On the contrary, CGT densities were similar on the two surfaces.

#### 2.3.2. Seed Germination Capacity and Morphometric Parameters

Data on seed germination capacity are presented in Table 2. The mean germination capacity ranged from 38 to 93%. The highest values were observed for SL and CV8, while CV7 showed the lowest value.

**Figure 4:**
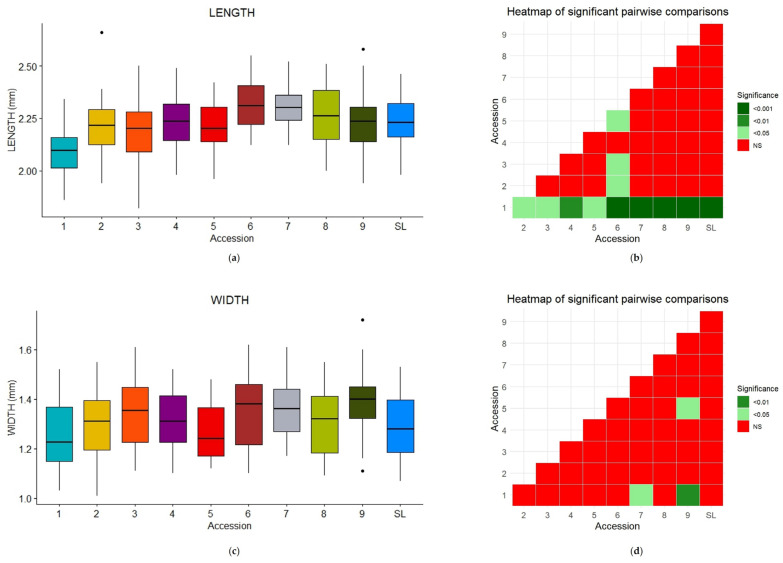
Boxplots of seed length (**a**); and width (**c**) for the nine old accessions (CVs) and SL. Heatmaps [(**b**) and (**d**), respectively] of the pairwise comparisons. Outliers are displayed as individual dots.

The mean values of seed widths and lengths are reported in Table S3 in the Supplementary Materials, together with the ANOVA results and the post hoc test *p*-values. A graphical representation of the distributions and the heatmap of significant pairwise comparisons are displayed in Figure 4.

As can be seen from both the boxplots and pairwise comparisons (Figure 4), the differences in seed lengths and widths were not as significant as in the case of trichomes.

**Figure 5:**
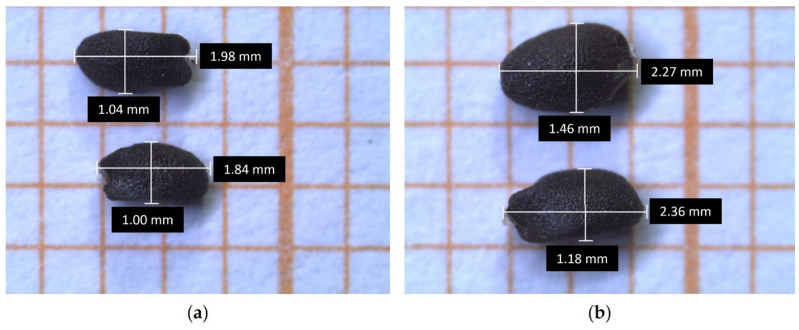
Seed length and width measurements: comparison between CV1 (**a**); and CV6 (**b**).

CV1 had the smallest length (2.10 ± 0.122 mm) (Table S3, Figure 4a,b and Figure 5a), but its width differed only from those of CV7 and CV9. CV6 showed the highest mean length value (2.32 ± 0.114 mm) (Table S3, Figure 5b), while CV9 had the highest mean width value (1.39 ± 0.124 mm) (Table S3). However, only a few significant comparisons were found (Figure 4).

### 2.4. Phytochemical Analysis

The main VOCs, which differed mostly among the CVs, and give basil a particular taste and flavor, are reported in Table 3. A complete list of the 112 VOCs identified in all basil samples can be seen in the Supplementary Materials (Table S4). Oxygenated monoterpenes were the most representative class in all samples (ranging from 49.42% in CV6 to 68.03% in CV1), followed by sesquiterpene hydrocarbons (from 22.12% in CV1 to 36.89% in CV7) (Table 4).

**Figure 6:**
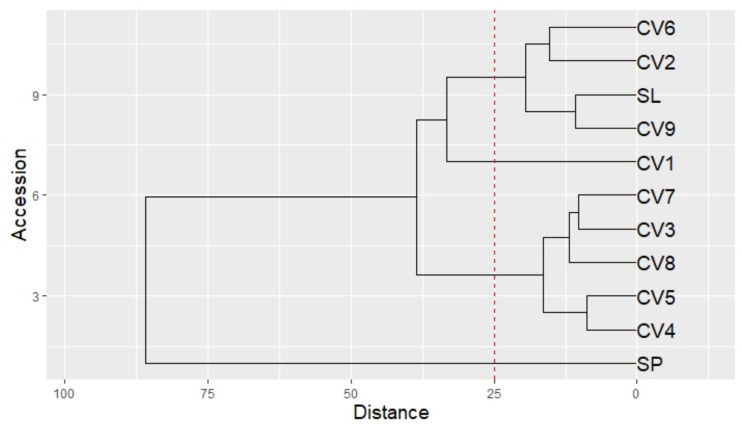
Dendrogram of the HCA performed on the complete composition of SPME microextraction of the leaves of CV1–9, SL, and SP. The dotted line cuts the dendrogram and forms the following four clusters: (1) SP; (2) CV1; (3) CV9, SL, CV2, CV6; and (4) CV4, CV5, CV8, CV3 and CV7.

The Hierarchal Cluster Analysis (HCA) highlighted the dominant effect of both genetic and pedo-climatic factors on the VOC profile. Four clusters were recognized (Figure 6): (1) SP; (2) CV1; (3) CV9, SL, CV2, CV6; and (4) CV4, CV5, CV8, CV3 and CV7. SP was completely separated from the old basil accessions (CV1–9) and from the SL.

Table 5 and Table 6 show the highest absolute differences in the aromatic composition between SP and the mean of all other accessions (CV1–9 and SL). SP was characterized by a considerable amount of methyl eugenol, β-farnesene and β-cubebene (Table 3, Table S4 and Table 5), while the other accessions (CV1–9 and SL) were richer in linalool, eugenol, and cis α-bergamotene (Table 3, Table S4 and Table 6). A difference in the camphor amount was also observed, with higher levels found in SP (Table 3, Table S4 and Table 5).

In addition, CV1 was categorized as a separate cluster with respect to the other Ligurian accessions (CV2–9 and SL), showing slightly higher levels of methyl-eugenol, eucalyptol, and camphor (Table 3 and Table S4), representing thus the closest accession to SP.

## 3. Discussion

The characterization of plants of food interest is extremely important to valorize products labelled with a PDO mark, showing typical intrinsic characteristics such as taste, color and aroma [8]. The high quality of these agrifood products is derived from the interaction between the cultivar used, the geographical environment, and the traditional methods of cultivation [7,8]. Therefore, reliable cultivar identification is fundamental for the protection of PDOs, ensuring the authenticity and traceability of these products [9,10].

Regarding *O. basilicum*, both morphological and phytochemical characterization of the different accessions/cultivars is crucial to correctly distinguish each specimen and thus manage a germplasm collection. Basil seeds are widely used in breeding programs to obtain plants with specific functional traits that allow for the selection of the most suitable cultivar for different applications, e.g., pharmacology, medicine, phytopathology, and horticulture [11,12]. Indeed, the cultivars currently used in Liguria to produce Genovese Basil PDO derive from old local accessions crossed over time with allochthonous genetic lines to obtain disease-resistant plants. Following this logic, plants of old accessions are still cultivated in order to preserve their seeds, which may become useful in the event of the spread of new pathogens that could cause severe losses of the current cultivars.

Plant phenotypes can be well identified by UPOV descriptors, since these characters are highly hereditable [13]. However, as the nine old accessions (CV1–9) and SL showed similar phenotypic characteristics, both micro-morphological and SPME analyses were necessary to accurately highlight the intra-specific variability.

For the micro-morphological characterization of basil, it is essential to assess the different types and densities of the secreting trichomes scattered on the leaf surface. These structures include peltate (PTGs) and capitate (CGTs) glandular trichomes [14,15]. PGTs represent the main site of essential oil biosynthesis and accumulation, while CGTs are mainly responsible for the synthesis of polysaccharides, proteins, and lipids [15,16]. The density and relative proportion of PGTs versus CGTs influence the essential oil content and composition; therefore, both types of trichomes contribute to the final aroma of basil [5].

Some cultivars, such as CV3 and CV4, were richer in PGTs. CV4 also showed a higher amount of CGTs on the lower surface, together with CV5. On the other hand, the cultivars that differed mostly from all the others in terms of lower trichome density were CV9 for PGTs and CV1 for CGTs. Trichome density can vary depending on plant genetic factors and growing environments [17]. However, since the old basil accessions were cultivated under the same conditions [4], it can be assumed that the differences in glandular trichome densities are linked to the plant’s genome. The presence of a high number of glandular trichomes, as found in CV4, can play an important ecological role, since they are important for the passive resistance of plants to pathogens and other biotic or abiotic stressors [17,18]. The different trichome densities support the variability in disease resistance among the commercial cultivars currently used to produce Genovese Basil PDO.

The intra-specific differences in seed germinability found among the accessions can have a genetic basis or derive from different sensitivities to environmental conditions or from the degree of dormancy [19]. Overall, considering that the old basil seeds were stored over the long term, the germination capacity was good, with only CV7 showing a germination capacity < 50%. The old accession CV8 showed a high percentage of germination, similar to that of the fresh seeds of the commercial cultivar SL. As seeds are living materials, viability testing through germination is an essential step for the optimal maintenance of a seed collection [19].

Seed morphometric data highlighted that only CV1 had significantly shorter seeds. The diversity of CV1 for phenotypical traits, trichome density and seed size agrees with the composition of its VOC profile, which showed greater similarity to SP than to the other Ligurian samples (CV2–9 and SL).

SL showed a strong similarity with CV7 and CV9 in terms of PGT density and with CV8 and CV9 with respect to CGT mean values. Additionally, the proximity to CV9 in the HCA of the VOC profiles suggests that SL could have been derived from this local old cultivar.

The different VOCs (e.g., terpenoids, alcohols, aldehydes, ketones and esters) that give basil its distinctive scent [20] have a strong impact on the consumer’s sensory perception [6]. Since the composition of VOCs can vary according to both genetic, environmental, and agricultural factors [21], the analytical profile of VOCs is relevant [6] to distinguish different accessions.

As was already observed for other samples of *O. basilicum* [11,22], oxygenated monoterpenes (e.g., linalool, eugenol, eucalyptol) and sesquiterpene hydrocarbons (e.g., cis α-bergamotene, β-cubebene, β-farnesene) were the most abundant volatiles in all the basil accessions under study. In addition, we observed a similar relative contribution of the different chemical classes to the VOCs composition. However, HCA analysis of the old Ligurian basil accessions (CV1–9) compared with SL and SP highlighted that the volatile fraction was affected by both the cultivar and the geographical area.

Methyl-chavicol (or estragole), commonly found in other sweet Italian basils (e.g., cv. Neapolitan) was absent in all samples, including SP, confirming that the cultivar is strictly associated with a particular aromatic profile [23]. This compound imparts a strong anise/liquorice aroma to basil [6], reducing the quality of this product. Genovese Basil PDO is instead characterized by a preponderance of linalool and the absence of estragole [22].

Regarding SP, the considerable amount of cubebene and farnesene gives basil leaves an intense herb odor [24,25] that, together with the camphoraceous aroma due to the presence of camphor, is often less appreciated by the consumers. Moreover, the high levels of methyl-eugenol found are considered responsible for a clove/minty aroma. It has been highlighted that high amounts of this compound could be of toxicological concern to human health, due to its suspected carcinogenic action [15,26]. In addition, it should be underlined that the absence of the minty aroma is one of the prerogatives of fresh Genovese Basil PDO, according to the PDO specifications [4].

The presence of off flavors, such as hay-like and earth odors, together with a mentholated aroma, has also been described as a negative consequence of the basil drying process, which affects, to a certain degree, the typical aroma of the fresh plant [27].

Among the old basil accessions, only CV1 showed slightly higher concentrations of methyl eugenol and camphor, resulting in the closest accession to SP. Therefore, the different traits revealed by the morphological analyses match the different compositions of the volatile compounds emitted by the essential oil.

The other clusters (CV2–9 and SL) presented instead an abundance of linalool, eugenol and cis α-bergamotene, which contribute to a fruity and sweet aroma, typical of Genovese Basil PDO. In particular, the presence of bergamotene can be a marker of basil cultivated in Liguria. This fact has been previously reported by a study [28] showing that this component, although in very low percentages, can be considered a distinctive characteristic of pesto sauce prepared with Genovese Basil PDO.

Essential oils are typified by major compounds that lead to specific odors (VOCs) [29]. Volatile Organic Compounds represent important quality markers and their qualitative and quantitative differences have a pivotal role in gastronomy and consumer appreciation [22,30]. Indeed, consumers choose which basil plants to purchase by smelling the leaves and looking for the aroma that is closest to their “olfactory memory”. The aroma arises from interactions between VOCs, which are influenced by numerous factors, including the cultivar and geographical origin. Therefore, consumers can distinguish PDO basil grown in Liguria from other plants cultivated in other regions by its distinctive aroma.

## 4. Materials and Methods

### 4.1. Plant Material, Soil Characteristics and Growing Methods

The nine old basil accessions were recovered from the following different areas in Liguria: 2 in Genoa (GE), 1 in Albenga (SV), 1 in Andora (SV), and 5 in the Dianese zone (IM).

Samples of these accessions were provided by CeRSAA, who cultivated the plants from seeds.

An adequate number of seeds for each variety were placed in seed trays to produce at least 100 basil plants per accession. The germination substrate used was chosen from those commonly used for this purpose (100% black peat). The trays were placed on benches in a greenhouse with controlled temperature and air humidity (minimum T 20 °C; maximum T 24 °C; RH 75–85%). The substrate temperature was not controlled and the lighting was the natural light available in the greenhouse.

At the phenological stage of two fully expanded leaves, plants were removed from the seed trays and transplanted into final pots (18 cm diameter). The growth substrate was common Ligurian soil, following PDO specifications [4]. To characterize the chemical and physical properties of this soil, a sample (about 200 g) was taken at a depth of about 20 cm. Then, it was air-dried at room temperature (RT) for three weeks, passed through a 2 mm sieve to remove gravel and debris, and sent to the soil testing laboratory “Regional Soil Analysis Laboratory in Sarzana” (La Spezia, Italy) (ISO 9001 certified). Routine laboratory analyses were performed in compliance with the proposed official Italian methods [31].

**Figure 7:**
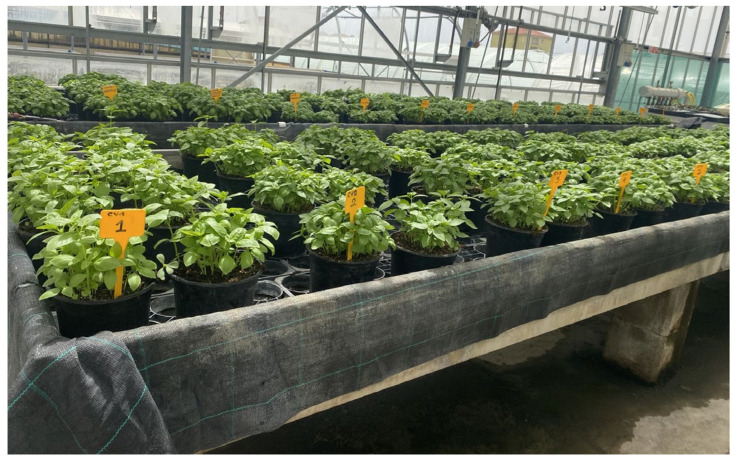
Greenhouse cultivation of old basil accessions (CV1–9) and SL at CeRSAA.

Plant nutrition was ensured by incorporating, at the time of pot filling, a controlled-release fertilizer with a formulation of N:P_2_O_5_:K_2_O = 15:15:15 at 2 g/L of substrate (released over 12 months at 20 °C). The pots were placed on raised benches in a greenhouse with controlled temperature and air humidity (minimum T 20 °C; maximum T 24 °C; RH 75–85%) (Figure 7). The substrate temperature was not controlled and the lighting was the natural light available in the greenhouse.

At the end of the initial root establishment phase and the first growth of the aerial parts of the plants, two successive pruning sessions were carried out to promote the tillering of the young plants.

To prevent infection and the spread of pathogens and pests that could affect the plants under the described cultivation conditions (such as *Peronospora belbahrii*, *Botrytis cinerea*, aphids, and thrips), authorized fungicides and insecticides were applied to the crop. Leaf sampling was carried out at the end of the safety period required for the applied plant protection products.

### 4.2. Phenotypic Traits

The nine old accessions and SL (Figure 7) were scored according to a standardized descriptor list developed by the International Union for the Protection of New Varieties of Plants (UPOV) [32]. The following phenotypic traits were studied: plant habit; plant height; leaf blade length; leaf blade length/width ratio; leaf blade shape; leaf blade blistering; leaf blade intensity of green color; leaf blade profile in cross-section; leaf blade orientation; and petiole pigmentation.

### 4.3. Micro-Morphological Analysis

#### 4.3.1. Leaves

**Figure 8:**
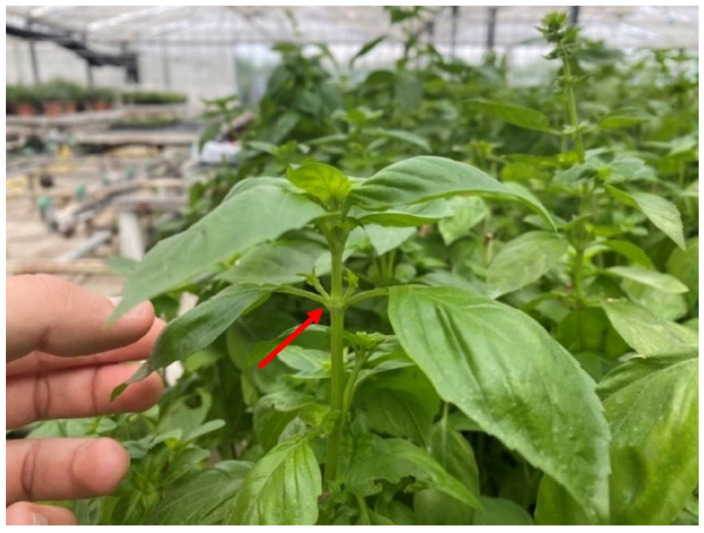
The third node (arrow) of a basil plant, from which leaf samples were collected.

For each accession, three plants were randomly selected and two fresh adult leaves were sampled from the third node [5,33] of each plant (Figure 8), for a total of six leaves per accession. The third node was chosen because, during harvesting, only the apical part of the plant, which is the most tender and fragrant, is cut off [3].

**Figure 9:**
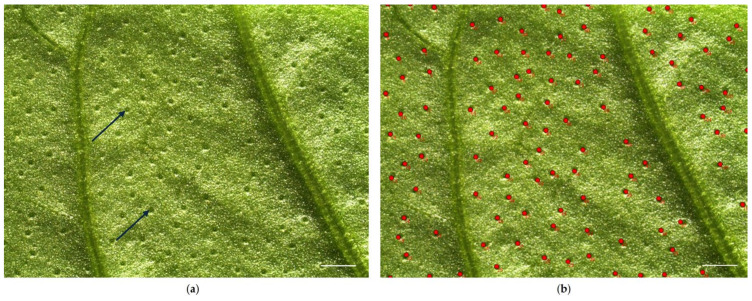
Lower surface of basil leaf: (**a**) a field of study, with clearly visible PGTs appearing as round, translucent vesicles partially sunken in the epidermis (arrows); and (**b**) PGT count on the same field (each trichome is marked with a red dot).

Leaves were then observed under a stereomicroscope (LEICA M205 C, Leica Microsystems, Wetzlar, Germany) to acquire images useful for calculating peltate glandular trichome (PGTs) densities on both leaf surfaces. The following magnifications were used: 1.25× for the lower page (Figure 9), which is richer in PGTs; and 2.5× for the upper page, which is generally characterized by a lower density of PGTs, making their identification more difficult. The analysis was conducted on the median zone of the leaf blade, considering 12 random fields within the area of investigation (six for the lower surface and six for the upper one), for a total of 72 micrographs per accession (36 for the lower surface and other 36 for the upper one). PGT density was estimated as the number of peltate glandular trichomes per unit leaf surface area (n/mm^2^). The areas were 85 mm^2^ for observation at 1.25× and 20.5 mm^2^ at 2.5×.

**Figure 10:**
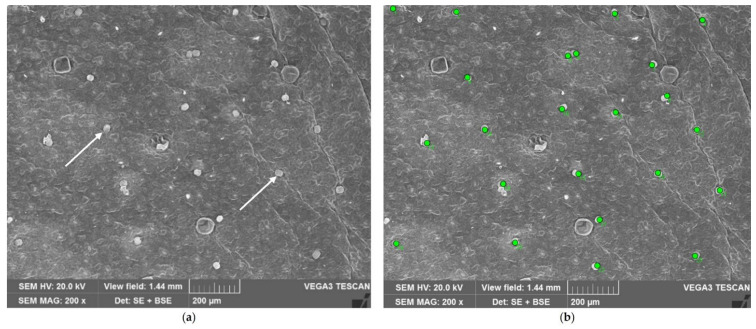
Lower surface of basil leaf: (**a**) a field of study, with small CGTs (arrows) spread among PGTs; and (**b**) CGT count on the same field (each trichome is marked with a green dot).

Capitate glandular trichomes (CGTs) are smaller than PGTs and thus are not as visible under stereomicroscopy. For this reason, CGT density was estimated using a Scanning Electron Microscope. Small portions of the median zone of the leaves were fixed in a FineFIX working solution (Milestone SRL, Sorisole, Bergamo, Italy), left overnight at 4 °C [34] and then dehydrated through a graded series of ethanol (70, 80, 90, and 100%). After being critical point-dried in CO_2_ (CPD, K850 2M Strumenti s.r.l., Rome, Italy), the specimens were mounted on aluminum stubs, covered with a 10 nm layer of gold and observed under a VEGA3-Tescan-type LMU microscope (Apollo, Tescan USA Inc., Cranberry Twp, PA, USA), operating at an accelerating voltage of 20 kV. Images were acquired at a magnification of 200× (Figure 10). The count was conducted on 24 random fields per accession (12 for the lower surface and other 12 for the upper one), distinguishing between marginal zones and midvein zones. CGT density was estimated as the number of capitate glandular trichomes per unit leaf surface area (n/mm^2^). The area was 1.55 mm^2^.

All the captured images were analyzed using the image processing software ImageJ (v 1.53t) [35].

#### 4.3.2. Seeds Germinability and Morphometric Parameters

Since the old basil seeds came from a collection stored at 5 °C, a preliminary check was conducted to assess their germinability. In order to make a comparison, SL seeds were also included in the analysis. In the laboratory, 30 seeds from each accession, divided into three replications, were placed in Petri dishes containing a bibulous paper disc with a diameter matching the dish. The dishes with the seeds were then incubated at 22–24 °C for a period of 7 days. At the end of this period, the correctly germinated seeds were counted to assess the germination capacity, i.e., the percentage of germinated seeds on the total tested seeds.

**Figure 11:**
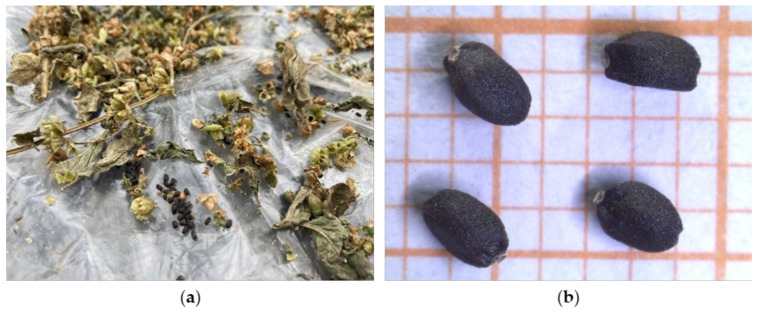
(**a**) Cleaning of the old basil accessions seeds; and (**b**) detail of the seeds under the stereomicroscope (CV4).

The morphometric parameters were detected on 30 seeds per accession. Seeds were manually cleaned to remove impurities (vegetal fragments, stones, soil residues, etc.) (Figure 11a). Then, their lengths and widths were measured in millimeters (mm) using LAS EZ (Leica Application Suite, version 1.6.0, Leica Microsystems) (Figure 11b).

### 4.4. Phytochemical Analysis

To identify and quantify the compounds that characterize basil aroma, Headspace Analysis by SPME/GC-MS was used. This is a fast, solvent-free method to determine the volatile constituents of essential oils [19]. Fresh basil leaves were chopped and a representative portion of 2 g was loaded into a specific SPME vial containing 8 mL of a saturated salt solution (NaCl). NaCl was used to increase the release of VOCs in the extraction headspace. The vials were then placed on a heated plate at 70 °C for 15 min. The SPME fiber (100 μm PDMS) was inserted into the headspace of the vial for 15 min to absorb the volatile compounds. After the extraction, the SPME fiber was injected into the injection port of the Agilent GC 6890N coupled with an Agilent 5973GC mass detector (Agilent technologies, Santa Clara, CA, USA). A TG-5SILMS capillary column (30 m × 250 μm × 0.25 μm) was used for the chromatographic separation (Agilent technologies, Santa Clara, CA, USA). The column temperature was set as follows: 50 °C for 2 min; then increased to 230 °C at a rate of 5 °C/min; and finally increased to 230 °C where it remained for an additional 10 min. The total analysis time was 48 min. The detector was operated under an electron impact (EI) ionization mode of 70 eV. The data acquisition was set within an m/z range of 33–260 atomic mass units (amu) with an immediate acquisition after the start.

VOCs were identified by comparing the retention index (RI) and the mass spectra with the available databases of those from the National Institute Standards and Technology (NIST) 2005 Mass Spectral Library. Quantification was performed by comparing the areas of the detected peaks with those of the authentic reference standards.

### 4.5. Statistical Analysis

Data obtained from the PGT count and seed measurements were analyzed by a one-way parametric Analysis of Variance (ANOVA), followed by pairwise comparisons (post hoc Tukey tests), since the sample size was sufficiently large (n = 36 and n = 30, respectively, per accession). CGT densities were estimated considering a smaller sample (n < 30); therefore, data were analyzed by a one-way non-parametric Kruskal–Wallis test, followed by pairwise comparisons (post hoc Wilcox tests). Since the ANOVA and Kruskal–Wallis test do not specify where the differences between samples may lie, pairwise comparisons were performed to evaluate relationships between the accessions.

A nested two-way nonparametric ANOVA was also performed to find potential differences in CGT density in the midvein and marginal zones of the leaf.

Moreover, a Hierarchical Cluster Analysis (HCA) was carried out to group the nine old basil accessions, SL, and SP according to similarities in the aromatic profiles obtained using SPME. This analysis was used to reveal VOC variations among cultivars linked to the area of cultivation (Liguria and Piedmont Region). The HCA was performed using the Ward’s method and by considering absolute values (mg/kg).

All analyses were carried out with software R, version 4.3.2 (R Core Team 2022). The significance level was set at 0.05.

## 5. Conclusions

This study provides a comparative account of intra-specific diversity in the glandular trichome density and VOC composition of several old Ligurian basil accessions, from which Genovese Basil PDO originated. The high quality of this Ligurian crop of excellence is derived from the interaction between the cultivars used, the geographical environment, and the traditional methods of cultivation. Our data highlights the importance of both in situ and ex situ conservation of old basil accessions to preserve germplasm biodiversity and maintain the production of Genovese Basil PDO in the future.

Further actions include the greater participation of local growers in maintaining the cultivation of these old Ligurian basil accessions, thereby creating a dense network of biodiversity custodians.

## Figures and Tables

**Table 1:** Ligurian soil features.

Soil Features	Unit	Value
Sand	g/kg	606
Silt	g/kg	250
Clay	g/kg	144
pH	-	8.1
Total N	g/kg	2.46
Total CaCO_3_	g/kg	145.6
Active CaCO_3_	g/kg	7
O.M.	g/kg	37.2
C/N	-	8.8
Cond.	1:5 dSm^−1^	0.21
C.E.C.	meq/100 g	9.5
Exch Ca	meq/100 g	7.66
Exch Mg	meq/100 g	1.37
Exch K	meq/100 g	0.47
Exch Na	meq/100 g	0.23

O.M.: organic matter; Cond.: electrical conductivity; C.E.C.: cation exchange capacity; Exch: exchangeable.

**Table 2:** Germination capacity (expressed as percentage) of CV1–9 and SL, shown as mean value ± SD.

Accession	Germination Capacity (%)
CV1	62 ± 3
CV2	88 ± 6
CV3	70 ± 0
CV4	83 ± 6
CV5	78 ± 8
CV6	85 ± 5
CV7	38 ± 10
CV8	93 ± 3
CV9	68 ± 10
SL	92 ± 6

**Table 3:** Main VOCs identified in CV1–9, SL and SP, expressed as mg/kg.

Compound	CV1	CV2	CV3	CV4	CV5	CV6	CV7	CV8	CV9	SL	SP
Eugenol	58.502	32.373	39.069	51.415	46.784	39.753	39.476	44.127	44.153	50.986	37.714
Linalool	32.212	29.420	37.789	38.150	40.918	22.752	32.835	40.046	28.374	25.019	13.202
Eucalyptol	27.905	16.228	18.944	15.921	14.961	12.295	15.231	16.262	15.320	14.191	16.965
cis α-Bergamotene	13.544	14.097	18.156	20.850	18.134	12.177	23.711	18.463	13.481	19.874	11.610
Methyl Eugenol	18.151	6.770	4.903	5.415	2.306	2.446	1.380	2.137	9.212	11.590	54.448
β-Cubebene	1.227	1.867	5.593	5.192	4.963	6.999	7.064	9.616	4.417	6.092	11.817
β-Farnesene	6.976	3.580	8.093	6.470	1.642	3.744	5.776	5.919	6.010	6.371	14.333
Camphor	2.541	0.864	1.792	1.704	1.015	1.164	1.159	1.047	1.182	1.244	3.531

**Table 4:** Relative abundance (%) of the different chemical classes of the VOCs found in CV1–9, SL, and SP.

Class	CV1	CV2	CV3	CV4	CV5	CV6	CV7	CV8	CV9	SL	SP
Monoterpene hydrocarbons	2.91	3.50	2.40	1.91	1.92	2.32	1.70	1.74	1.61	1.64	1.22
Oxygenated monoterpenes	68.03	64.42	54.71	58.03	60.46	49.42	51.04	50.13	63.22	58.24	56.13
Sesquiterpene hydrocarbons	22.12	25.37	33.50	30.84	28.18	33.24	36.89	33.90	26.23	30.56	31.91
Oxygenated sesquiterpenes	5.07	4.21	7.51	6.64	6.94	10.78	7.13	9.99	6.48	6.66	6.77
Others	0.91	1.42	1.01	1.20	1.46	2.57	2.14	2.58	1.76	2.10	1.38

**Table 5:** Comparison between SP and all other accessions (CV1–9 and SL). The highest positive values (delta abs) are due to higher quantities of the listed compounds in SP.

	SP	Mean Others	Delta Abs
Methyl eugenol	54.448	6.431	48.017
β-Farnesene	14.333	5.458	8.875
β-Cubebene	11.817	5.303	6.515
β-Patchoulene	6.232	3.012	3.220
α-Bulnesene	5.563	2.630	2.932
(-)-β-Elemene	5.678	3.005	2.673
Camphor	3.531	1.371	2.160
α-Humulene	4.478	2.709	1.769

**Table 6:** Comparison between SP and all other accessions (CV1–9 and SL). The highest values with a negative sign (delta abs) are due to lower quantities of the listed compounds in SP.

	SP	Mean Others	Delta Abs
Linalool	13.202	32.751	−19.549
Eugenol	37.714	44.664	−6.949
cis α-Bergamotene	11.610	17.249	−5.639
Isocaryophyllene	0.000	1.518	−1.518
τ-Cadinene	4.408	5.803	−1.395
β-Gurjunene	0.000	0.677	−0.677
β-Myrcene	0.508	1.097	−0.589
α-Ionene	0.254	0.776	−0.522

## Data Availability

The data presented in this study are available in the article/Supplementary Materials.

## Supplementary Materials

The following supporting information can be downloaded at: https://www.mdpi.com/article/10.3390/plants14040553/s1.
