# Supplementary material for: Morphological and Phytochemical Characterization of Old Ligurian Basil Accessions: Recovery of Old Biodiversity for Future Exploitation
Source: Plants (Basel). 2025 Feb 11;14(4):553. doi: 10.3390/plants14040553 (PMC11859914; doi:10.3390/plants14040553)
Supplement: Supplementary file 1 [file plants-14-00553-s001.zip › plants-3393717-supplementary.pdf]

Table S1. PGTs density of the nine old accessions (CVs) and SL, shown as mean value ± SD, for both lower and upper surface.

Accession (CV)	PGTs density (n/mm²)	
	**Lower surface**	**Upper surface**
	**Mean ± SD**	**Mean ± SD**
1	103.0 ± 14.0	23.0 ± 3.09
2	121.0 ± 44.7	25.6 ± 5.16
3	141.0 ± 24.9	27.3 ± 4.77
4	205.0 ± 52.3	27.8 ± 5.99
5	97.0 ± 20.6	13.1 ± 2.14
6	114.0 ± 21.9	17.1 ± 2.84
7	80.6 ± 12.8	13.7 ± 2.61
8	95.4 ± 13.6	17.2 ± 3.71
9	59.6 ± 12.8	9.5 ± 2.36
SL	68.2 ± 36.4	10.2 ± 3.81
ANOVA F-value	75.72	119.2
ANOVA p-value	<0.0001	<0.0001

p-values of the post-hoc pairwise significant comparisons (lower surface) in ascending order: 1 vs. 3 (<0.0001), 1 vs 4 (<0.0001), 1 vs. 9 (<0.0001), 1 vs. SL (<0.0001), 2 vs. 4 (<0.0001), 2 vs. 7 (<0.0001), 2 vs. 9 (<0.0001), 2 vs. SL (<0.0001), 3 vs. 4 (<0.0001), 3 vs. 5 (<0.0001), 3 vs. 7 (<0.0001), 3 vs. 8 (<0.0001), 3 vs. 9 (<0.0001), 3 vs. SL (<0.0001), 4 vs. 5 (<0.0001), 4 vs. 6 (<0.0001), 4 vs. 7 (<0.0001), 4 vs. 8 (<0.0001), 4 vs. 9 (<0.0001), 4 vs. SL (<0.0001), 5 vs. 9 (<0.0001), 6 vs. 7 (<0.0001), 6 vs. 9 (<0.0001), 6 vs. SL (<0.0001), 8 vs. 9 (<0.0001), 5 vs. SL (0.0011), 8 vs. SL (0.0030), 3 vs. 6 (0.0047), 2 vs. 8 (0.0062), 2 vs. 5 (0.0144), 1 vs. 7 (0.0299).

p-values of the post-hoc pairwise significant comparisons (upper surface) in ascending order: 1 vs. 4 (<0.001), 1 vs. 5 (<0.0001), 1 vs. 6 (<0.0001), 1 vs. 7 (<0.0001), 1 vs. 8 (<0.0001), 1 vs. 9 (<0.0001), 1 vs. SL (<0.0001), 2 vs. 5 (<0.0001), 2 vs. 6 (<0.0001), 2 vs. 7 (<0.0001), 2 vs. 8 (<0.0001), 2 vs. 9 (<0.0001), 2 vs. SL (<0.0001), 3 vs. 5 (<0.0001), 3 vs. 6 (<0.0001), 3 vs. 7 (<0.0001), 3 vs. 8 (<0.0001), 3 vs. 9 (<0.0001), 3 vs. SL (<0.0001), 4 vs. 5 (<0.0001), 4 vs. 6 (<0.0001), 4 vs. 7 (<0.0001), 4 vs. 8 (<0.0001), 4 vs. 9 (<0.0001), 4 vs. SL (<0.0001), 5 vs. 6 (<0.0001), 6 vs. 9 (<0.0001), 6 vs. SL (<0.0001), 8 vs. 9 (<0.0001), 8 vs. SL (<0.0001), 1 vs. 3 (0.0001), 5 vs. 8 (0.0003), 7 vs. 9 (0.0003), 5 vs. 9 (0.0047), 7 vs. SL (0.0047), 7 vs. 8 (0.0053), 6 vs. 7 (0.0083).

Table S2. CGTs density of the old nine accessions (CVs) and SL, shown as mean value ± SD, for both lower and upper surface.

**Accession (CV)**	**CGTs density (n/mm²)**	
	Lower surface	Upper surface
	Mean ± SD	Mean ± SD
1	8.5 ± 1.78	4.08 ± 0.669
2	9 ± 2.63	8.92 ± 2.15
3	13.3 ± 7.60	13.7 ± 8.22
4	20 ± 5.92	11.9 ± 3.29
5	15 ± 4.26	11 ± 3.62
6	8.58 ± 3.60	8.5 ± 2.28
7	10 ± 3.74	9.75 ± 3.39
8	7.58 ± 2.07	6.75 ± 1.60
9	7.83 ± 3.10	7.5 ± 3.71
SL	7.25 ± 1.22	7.75 ± 1.29
Kruskal-Wallis statistic	46.717	45.072
Kruskal-Wallis p-value	<0.0001	<0.0001

p-values of the post-hoc pairwise significant comparisons (lower surface) in ascending order: 1 vs. 4 (0.0006), 4 vs. 8 (0.0006), 4 vs. 9 (0.0006), 4 vs. SL (0.0006), 5 vs. SL (0.0006), 2 vs. 4 (0.0008), 5 vs. 8 (0.0008), 1 vs. 5 (0.0012), 4 vs. 6 (0.0013), 5 vs. 9 (0.0018), 4 vs. 7 (0.0021), 2 vs. 5 (0.0044), 5 vs. 6 (0.0068), 5 vs. 7 (0.0378).
p-values of the post-hoc pairwise significant comparisons (upper surface) in ascending order: 1 vs. 2 (0.0003), 1 vs. 4 (0.0003), 1 vs. 5 (0.0003), 1 vs. SL (0.0003), 1 vs. 6 (0.0004), 1 vs. 7 (0.0004), 1 vs. 8 (0.0005), 1 vs. 3 (0.0028), 4 vs. 8 (0.0028), 4 vs. SL (0.0102), 5 vs. 8 (0.0135), 4 vs. 9 (0.0401), 4 vs. 6 (0.0434), 2 vs. 8 (0.0494).

Table S3. Length and width of the seeds of the nine old accessions (CVs) and SL, shown as mean value ± SD.

Accession (CV)	Seeds	
	**Length (mm)**	**Width (mm)**
	**Mean ± SD**	**Mean ± SD**
1	2.10 ± 0.122	1.25 ± 0.137
2	2.22 ± 0.137	1.30 ± 0.134
3	2.20 ± 0.156	1.35 ± 0.137
4	2.23 ± 0.114	1.32 ± 0.112
5	2.21 ± 0.114	1.27 ± 0.115
6	2.32 ± 0.114	1.34 ± 0.143
7	2.31 ± 0.097	1.36 ± 0.124
8	2.26 ± 0.134	1.31 ± 0.138
9	2.24 ± 0.147	1.39 ± 0.124
SL	2.24 ± 0.119	1.29 ± 0.127
ANOVA F-value	7.220	3.245
ANOVA p-value	<0.0001	0.0009

p-values of the post-hoc pairwise significant comparisons (length) in ascending order: 1 vs. 6 (<0.0001), 1 vs. 7 (<0.0001), 1 vs. 8 (<0.0001), 1 vs. 9 (0.0007), 1 vs. SL (0.0009), 1 vs. 4 (0.0027), 1 vs. 2 (0.0113), 3 vs. 6 (0.0121), 1 vs. 5 (0.0183), 5 vs. 6 (0.0216), 1 vs. 3 (0.0319), 2 vs. 6 (0.0340).
p-values of the post-hoc pairwise significant comparisons (width) in ascending order: 1 vs. 9 (0.0014), 5 vs. 9 (0.0121), 1 vs. 7 (0.0464).

Table S4. VOCs identified in CV1-9, SL and SP, expressed as mg/kg. The chemical compounds which differed mostly among CVs are showed in bold.

Compound	CV1	CV2	CV3	CV4	CV5	CV6	CV7	CV8	CV9	SL	SP
(+)-Aromadendrene	0.538	0.390	0.689	0.690	0.553	0.607	0.938	0.983	0.427	0.446	0.785
(+)-Epi-bicyclosesquiphellandrene	0.000	0.306	0.906	0.669	0.756	1.029	0.754	1.218	0.542	0.650	0.946
(+)-trans-Chrysanthenyl Acetate	0.000	0.000	0.000	0.004	0.004	0.003	0.004	0.004	0.003	0.019	0.000
(-)-Myrtenyl acetate	0.009	0.014	0.020	0.020	0.018	0.017	0.018	0.021	0.016	0.019	0.000
1-(2-Furyl)-2-pentanone	0.022	0.014	0.039	0.020	0.018	0.017	0.018	0.009	0.016	0.004	0.000
1,2-Dihydrolinalool	0.043	0.042	0.059	0.061	0.074	0.051	0.037	0.064	0.049	0.037	0.000
2-Hydroxycineol	0.000	0.000	0.000	0.000	0.000	0.000	0.000	0.000	0.000	0.000	0.023
2-Oxo-1,8-cineole	0.043	0.028	0.039	0.020	0.037	0.017	0.037	0.043	0.033	0.037	0.069
2,4-Hexadienal	0.022	0.014	0.020	0.020	0.000	0.000	0.000	0.009	0.005	0.000	0.000
3-Carene	0.129	0.056	0.098	0.061	0.092	0.067	0.037	0.085	0.082	0.093	0.115
3-Octanol	0.000	0.000	0.000	0.000	0.000	0.202	0.037	0.085	0.066	0.074	0.069
3-Octenol	0.301	0.348	0.217	0.081	0.111	0.236	0.276	0.256	0.378	0.223	0.669
3-Octanone	0.000	0.042	0.177	0.101	0.129	0.000	0.092	0.299	0.164	0.037	0.231
3-Thujene	0.086	0.070	0.079	0.061	0.055	0.051	0.037	0.043	0.049	0.056	0.069
3,7-Dimethyl-1-octene	0.000	0.000	0.000	0.000	0.000	0.000	0.000	0.000	0.000	0.093	0.000
4-Terpineol	1.184	0.279	0.650	0.243	0.683	0.101	0.074	0.043	0.230	0.093	0.000
Acoradiene	0.129	0.139	0.295	0.203	0.129	0.270	0.258	0.278	0.131	0.186	0.000
Borneol	0.172	0.460	0.236	0.649	0.553	0.489	0.405	0.192	0.673	0.483	0.000
Bornyl Acetate	1.615	0.000	0.000	0.000	0.000	0.000	0.000	0.000	0.000	0.000	0.000
Camphene	0.043	0.084	0.098	0.061	0.055	0.051	0.037	0.043	0.049	0.074	0.092
**Camphor**	2.541	0.864	1.792	1.704	1.015	1.164	1.159	1.047	1.182	1.244	3.531
Caryophyllene	0.861	0.529	0.807	0.710	0.572	0.826	0.699	1.133	0.640	0.762	1.846
Chavicol	0.043	0.028	0.020	0.041	0.018	0.017	0.018	0.043	0.016	0.037	0.023
cis 3-Hexen-1-ol	0.000	0.000	0.138	0.000	0.166	0.000	0.000	0.000	0.000	0.000	0.069
**cis α-Bergamotene**	13.544	14.097	18.156	20.850	18.134	12.177	23.711	18.463	13.481	19.874	11.610
cis-allo-Ocimene	0.022	0.028	0.039	0.041	0.037	0.051	0.055	0.000	0.049	0.019	0.000
cis-Geraniol	0.000	0.000	0.000	0.000	0.000	0.000	0.000	0.000	0.000	0.019	0.000
cis-β-Ocimene	2.627	2.452	3.072	2.799	1.992	2.479	2.115	2.222	1.741	1.932	2.285
cis Methyl p-methoxycinnamate	0.108	0.000	0.000	0.000	0.000	0.000	0.000	0.000	0.000	0.093	0.069
cis-Pinen-3-ol	0.000	0.000	0.039	0.020	0.018	0.017	0.018	0.021	0.000	0.019	0.023
m-cymene	0.043	0.028	0.039	0.020	0.055	0.034	0.018	0.000	0.016	0.037	0.023
Copaene	0.000	0.000	0.000	0.000	0.000	0.000	0.000	0.000	0.000	0.000	0.000
Cubenol	0.775	0.432	0.827	0.974	0.922	1.349	0.865	1.624	0.772	0.836	0.923
Eremophylene	0.000	0.000	0.000	0.000	0.000	0.000	0.000	0.000	0.000	0.000	0.000
**Eucalyptol**	27.905	16.228	18.944	15.921	14.961	12.295	15.231	16.262	15.320	14.191	16.965
**Eugenol**	58.502	32.373	39.069	51.415	46.784	39.753	39.476	44.127	44.153	50.986	37.714
Farnesol	0.237	0.042	0.059	0.041	0.055	0.051	0.037	0.043	0.066	0.074	0.046
Fenchyl acetate	0.000	0.028	0.000	0.000	0.000	0.000	0.000	0.000	0.000	0.000	0.000
Fenchone	0.000	0.167	0.000	0.000	0.000	0.000	0.000	0.000	0.000	0.000	0.000
Elixene	0.172	0.084	0.217	0.162	0.148	0.270	0.110	0.214	0.099	0.111	0.000
exo-2-Hydroxycineole acetate	0.172	0.070	0.118	0.101	0.111	0.101	0.018	0.085	0.066	0.111	0.023
Eudesm-7(11)-en-4-ol	0.215	0.070	0.059	0.041	0.074	0.067	0.055	0.064	0.049	0.074	0.162
Hexanal	0.043	0.056	0.059	0.061	0.055	0.034	0.074	0.064	0.066	0.056	0.046
Himachala-2,4-diene	0.624	0.209	0.197	0.487	0.351	0.540	0.570	0.427	0.246	0.390	0.785

Isoborneol	0.000	0.000	0.000	0.000	0.000	0.000	0.000	0.000	0.000	0.019	0.000
Isobornyl acetate	0.000	0.000	0.000	0.000	0.000	0.000	0.000	0.000	0.000	0.019	0.000
Isocaryophyllene	1.077	0.683	2.245	1.663	1.771	1.400	1.931	1.239	1.182	1.987	0.000
Isoeugenol	0.495	0.376	0.433	0.527	0.351	0.472	0.386	0.342	0.476	0.371	0.323
Isolimonene	0.000	0.000	0.000	0.000	0.000	0.000	0.000	0.000	0.016	0.000	0.000
Isoterpinolene	0.474	0.237	0.276	0.264	0.166	0.236	0.166	0.150	0.148	0.130	0.138
(+)-Ledene	1.787	1.100	2.225	1.055	0.830	1.349	1.232	2.244	0.443	0.594	0.969
Limetol	0.009	0.003	0.020	0.061	0.007	0.017	0.018	0.000	0.016	0.000	0.000
Limonene	1.034	0.836	0.197	0.183	0.406	0.759	0.423	0.085	0.066	0.074	0.069
**Linalool**	32.212	29.420	37.789	38.150	40.918	22.752	32.835	40.046	28.374	25.019	13.202
Linalylanthranilate	0.011	0.014	0.020	0.020	0.018	0.017	0.018	0.000	0.005	0.019	0.023
Methyl Cinnamate	0.237	0.306	0.197	0.183	0.332	0.034	0.074	0.107	0.213	0.260	0.046
**Methyl Eugenol**	18.151	6.770	4.903	5.415	2.306	2.446	1.380	2.137	9.212	11.590	54.448
Nerolidol	0.237	0.098	0.236	0.203	0.258	0.405	0.221	0.449	0.213	0.204	0.577
Octanal	0.151	0.098	0.138	0.101	0.037	0.000	0.055	0.128	0.099	0.074	0.115
n-Octylacetate	0.022	0.028	0.039	0.061	0.037	0.067	0.037	0.021	0.033	0.056	0.000
Oxime methoxy phenyl	0.000	0.000	0.000	0.000	0.000	0.000	0.037	0.000	0.000	0.000	0.000
Pinocarvone	0.022	0.014	0.059	0.041	0.018	0.017	0.037	0.043	0.016	0.019	0.046
Sabinene	0.301	0.320	0.394	0.264	0.387	0.219	0.313	0.534	0.279	0.316	0.346
Spathulenol	0.000	0.000	0.000	0.000	0.000	0.000	0.000	0.000	0.000	0.019	0.023
trans-2-Hexenal	0.194	0.293	0.197	0.304	0.258	0.169	0.497	0.278	0.164	0.409	0.231
trans-3-Hexen-1-ol	0.172	0.237	0.098	0.466	0.129	0.152	0.092	0.256	0.296	0.353	0.346
trans-3-Hexenyl Butyrate	0.000	0.000	0.000	0.000	0.000	0.034	0.000	0.000	0.000	0.000	0.000
trans-Geraniol	0.000	0.000	0.000	0.000	0.000	0.000	0.000	0.000	0.000	0.056	0.000
trans-α-Bergamotene	0.345	0.334	0.610	0.852	0.553	0.304	0.497	0.577	0.345	0.613	0.000
trans-β-Ocimene	0.000	0.000	0.000	0.000	0.000	1.467	1.619	1.389	0.969	0.947	0.831
trans Methyl p-methoxycinnamate	0.194	0.000	0.000	0.000	0.000	0.000	0.000	0.000	0.000	0.167	0.115
α-Amorphene	1.012	0.306	0.807	0.892	0.664	0.742	0.828	0.684	0.493	0.260	0.785
α-Bisabolene	0.151	0.111	0.118	0.203	0.111	0.169	0.166	0.085	0.066	0.111	0.115
α-Bisabolol	0.151	0.070	0.098	0.142	0.111	0.118	0.147	0.192	0.131	0.204	0.162
L-α-Bornyl acetate	0.000	0.947	1.418	1.379	1.254	1.855	0.736	0.705	1.067	1.374	0.577
α-Bulnesene	1.959	1.142	4.135	2.474	2.490	3.711	2.962	3.611	1.773	2.043	5.563
α-Citral	0.022	0.014	0.000	0.000	0.000	0.000	0.007	0.009	0.007	0.019	0.023
α-Copaene	0.215	0.111	0.354	0.203	0.000	0.287	0.110	0.342	0.115	0.204	0.000
α-Cubebene	0.689	0.376	0.788	0.548	0.646	0.742	0.552	0.855	0.378	0.520	0.739
α-Humulene	3.079	1.588	2.087	2.414	3.136	3.609	2.612	2.906	2.184	3.473	4.478
α-Ionene	0.409	0.376	0.610	0.629	0.941	1.467	0.589	1.560	0.197	0.984	0.254
α-Muurolene	0.560	0.237	0.473	0.548	0.369	0.742	0.497	0.641	0.279	0.204	0.462
α-Phellandrene	0.345	0.084	0.158	0.162	0.092	0.101	0.055	0.128	0.082	0.093	0.046
α-Pinene	0.280	0.362	0.354	0.203	0.240	0.236	0.221	0.299	0.246	0.297	0.369
α-Selinene	0.000	0.000	0.709	0.264	0.609	0.590	0.975	1.175	0.411	0.706	1.962
(R) α-Terpineol	0.517	0.237	0.217	0.000	0.424	0.169	0.074	0.150	0.345	0.371	0.415
(S) α-Terpineol	2.756	1.254	1.615	1.765	1.660	1.400	1.637	1.496	2.151	1.727	1.916
α-Terpinolene	0.624	0.529	0.571	0.588	0.351	0.405	0.368	0.342	0.312	0.334	0.439
L-β-Bisabolene	0.603	0.474	0.866	1.136	0.719	0.860	1.140	0.470	0.887	0.334	0.369
β-Bisabolol	0.000	0.042	0.039	0.061	0.055	0.067	0.037	0.085	0.033	0.037	0.069
β-Cadinene	2.261	1.114	0.945	1.582	1.347	2.108	1.288	0.962	0.706	0.929	1.823

β-Citral	0.000	0.000	0.000	0.000	0.000	0.000	0.000	0.000	0.000	0.019	0.000
β-Copaene	0.108	0.139	0.295	0.183	0.240	0.202	0.202	0.363	0.181	0.279	0.277
**β-Cubebene**	1.227	1.867	5.593	5.192	4.963	6.999	7.064	9.616	4.417	6.092	11.817
(+)-β-Elemene	0.409	0.125	0.354	0.284	0.369	0.506	0.313	0.513	0.213	0.223	0.485
(-)-β-Elemene	2.153	1.212	4.273	2.677	2.970	4.351	2.649	5.193	2.250	2.322	5.678
β-Eudesmol	0.581	0.195	0.295	0.406	0.332	0.489	0.331	0.598	0.296	0.409	0.600
**β-Farnesene**	6.976	3.580	8.093	6.470	1.642	3.744	5.776	5.919	6.010	6.371	14.333
β-Gurjunene	0.926	0.209	0.335	1.237	0.941	0.287	1.140	0.000	0.821	0.873	0.000
β-Himachalene	0.000	0.111	0.000	0.345	0.424	0.422	0.386	0.962	0.181	0.000	0.000
β-Myrcene	1.636	1.268	1.457	1.217	0.959	0.995	0.773	1.197	0.706	0.762	0.508
β-Patchoulene	1.507	1.240	4.391	2.698	3.173	3.795	2.888	5.364	2.003	3.065	6.232
(-)-β-Pinene	0.646	0.613	0.591	0.406	0.406	0.405	0.497	0.620	0.361	0.576	0.716
β-Sesquiphellandrene	0.840	0.418	1.122	1.217	1.181	0.523	1.656	1.026	0.772	0.984	0.623
β-Terpineol	0.129	0.139	0.295	0.162	0.314	0.169	0.258	0.321	0.378	0.316	0.300
δ-Cadinene	0.194	0.181	0.256	0.183	0.166	0.388	0.147	0.342	0.148	0.167	0.300
(-)-δ-Cadinol	0.108	0.042	0.079	0.122	0.074	0.152	0.074	0.150	0.082	0.111	0.115
τ-Cadinene	4.565	2.549	7.325	6.571	5.479	6.645	6.935	10.236	3.251	4.476	4.408
τ-Cadinol	4.091	2.173	3.840	5.395	4.206	6.139	3.955	8.419	4.286	4.736	4.893
τ-Gurjunene	0.431	0.376	1.634	0.710	1.107	2.176	1.619	1.774	0.394	0.464	1.131
τ-Muurolene	0.452	0.306	0.748	0.750	0.498	0.911	0.883	1.004	0.673	0.687	0.785
τ-Terpinene	0.754	0.446	0.512	0.446	0.369	0.422	0.221	0.278	0.328	0.297	0.000
**TOTAL**	215.32	139.30	196.92	202.82	184.48	168.66	183.95	213.69	164.20	185.74	230.81
